# Knowledge grounded medical dialogue generation using augmented graphs

**DOI:** 10.1038/s41598-023-29213-8

**Published:** 2023-02-27

**Authors:** Deeksha Varshney, Aizan Zafar, Niranshu Kumar Behera, Asif Ekbal

**Affiliations:** Department of Computer Science and Engineering, Indian Institute of Technology, Patna, Patna, 801103 India

**Keywords:** Computer science, Software

## Abstract

Smart healthcare systems that make use of abundant health data can improve access to healthcare services, reduce medical costs and provide consistently high-quality patient care. Medical dialogue systems that generate medically appropriate and human-like conversations have been developed using various pre-trained language models and a large-scale medical knowledge base based on Unified Medical Language System (UMLS). However, most of the knowledge-grounded dialogue models only use local structure in the observed triples, which suffer from knowledge graph incompleteness and hence cannot incorporate any information from dialogue history while creating entity embeddings. As a result, the performance of such models decreases significantly. To address this problem, we propose a general method to embed the triples in each graph into large-scalable models and thereby generate clinically correct responses based on the conversation history using the recently recently released MedDialog(EN) dataset. Given a set of triples, we first mask the head entities from the triples overlapping with the patient’s utterance and then compute the cross-entropy loss against the triples’ respective tail entities while predicting the masked entity. This process results in a representation of the medical concepts from a graph capable of learning contextual information from dialogues, which ultimately aids in leading to the gold response. We also fine-tune the proposed Masked Entity Dialogue (MED) model on smaller corpora which contain dialogues focusing only on the Covid-19 disease named as the Covid Dataset. In addition, since UMLS and other existing medical graphs lack data-specific medical information, we re-curate and perform plausible augmentation of knowledge graphs using our newly created Medical Entity Prediction (MEP) model. Empirical results on the MedDialog(EN) and Covid Dataset demonstrate that our proposed model outperforms the state-of-the-art methods in terms of both automatic and human evaluation metrics.

## Introduction

Health conversational assistants (or Chatbots) for patients act as a medical consultant, which usually provides simple and relevant measures to avoid infection from various diseases. They are designed to interact with patients in real time, inquiring about their medical issues and past records while also attempting to make useful suggestions. This enabled clinicians to use telemedicine, which is critical in pandemic circumstances where physical contact between patients and doctors is restricted. Chatbots are progressively being used to help people communicate in the open domain settings in order to exchange information^1–3^ and to assist professionals in completing a specific activity^4,5^. A medical dialogue system can serve as a physician’s assistant, inquiring about the patient’s medical, medication, social, personal, and family history, as well as a thorough review of symptoms and possibly a physical examination^6–8^. As a result, intelligent medical dialogue systems have the potential to reduce the workload of physicians.

More access to external sources of medical knowledge may aid in resolving the problem of limited semantic understanding during response generation. For example, to comprehend the utterance-response pair shown in Table 1, it is demonstrated how, rather than expecting a generic response, we might achieve the ground truth response by combining external medical information. Different weights should be assigned to different tail entities extracted from the triples acquired for the words *dry eyes* and *cataract* based on their presence in the gold response which have been linked using different relations. Tail entities, such as *blurring of vision*, *cornea* should certainly be given more importance due to their presence in the target response. Recently, Li et al.^9^ proposed a reasoning method over knowledge graphs for medical dialogue generation using a large-scale medical corpus to deliver appropriate medical responses, but they only learn local embeddings for the entities in different triples, ignoring the contextual information.

A medical knowledge graph is a multidimensional graph with nodes and edges, that represent the relationships between different medical concepts in several biomedical domains. However, the current medical graphs lack local information contained in medical dialogues i.e there is no representation of medical entities and relations with respect to the conversations. A team of subject-matter experts may be hired to map all of the connections between various treatments, illnesses, and other scientific concepts in order to address these issues. Unfortunately, the cost of hiring a group of medical experts to accomplish this task is rather high. In that instance, we may rapidly and simply extract those relations using NLP approaches.

**Table 1:** An example from the MedDialog(EN) dataset illustrating the lack of global medical knowledge in existing dialogue models.

Patient’s query	Will accidental entry of metal shavings into eyes lead to **dry eyes** and **cataract** ?
Doctor’s Response	Hi, **blurred vision** may be due to a corneal problem, cataract or retinal scars. After trauma, all these complications can occur. **Dry eyes** will improve with artificial tears and cornea will get normal. but any **corneal scar** or opacity will remain and can hinder in vision. cataract may be age-related or due to trauma. so if it is visually significant it is treated by surgical removal and intraocular lens implantation.
Generic Response	Hi! and thank you so much for this query.
Medical Knowledge Triples	*{cataract, co-occurs with, blurring of vision}, {cataract, evaluation of, cornea}, {cataract, co-occurs with, dry eyes}*

The generic response was observed during the inference stage by previous approaches.

In this study, we propose a novel method for medical dialogue generation called MED, to generate efficient responses by leveraging both the context information and relevant medical knowledge graphs using pre-trained language models. First, we build knowledge graphs using UMLS^10^ database and BERT-based entity prediction models as described in section “Construction of the medical entity graph”. Second, we employ our MED model to produce appropriate responses using these knowledge graphs. To be more specific, we mask the head entities from triples and use the masked sequence as the new input. The aim is to predict the tail entities that correspond to the masked head entities while also generating the response using the hidden states of the masked input sequence. In order to achieve this, we build models using the MedDialog(EN) corpus and the Covid Dataset for generating the appropriate responses. The proposed method performs well for knowledge graph enhanced medical dialogue generation.

Our current work makes the following contributions:

1. We propose a new medical-knowledge-aware neural model, *MED* for medical dialogue generation that uses large-scale pre-trained language models to incorporate the triples from the knowledge graph for generating medically relevant dialogues. By greatly boosting the knowledge representation using a large amount of information from dialogue context, this method investigates the learning of contextual entity embeddings.
2. We use the UMLS database to create a medical knowledge graph that is supplemented with several medical entities not discovered in the UMLS database and are extracted using BERT-based medical entity prediction models. This provides the language models with both global knowledge from UMLS as well as conversation-oriented medical entities from the medical datasets.
3. We show that our model is capable of integrating graph contextualized information into large-scale pretrained language models which outperforms the strong baselines on the MedDialog(EN) and Covid Dataset using extensive qualitative and quantitative evaluation.

The rest of the article is organised as follows. Some of the earlier efforts in this field are summarised in the “Related work” section. Then, we describe our proposed methodology for generating clinically accurate response. The dataset used for the suggested generative model is briefly described in the next section. We give a detailed account of the experimental evaluation in the following part, followed by a quick review of how well the suggested generative model performed. We finish the article by describing the originality of the work and its future applications.

## Related work

The first Chatbot, ELIZA^11^, used a keyword/pattern matching mechanism to find a pattern and the corresponding response for a given user text. Jia^12^ improved upon ELIZA by using textual knowledge and reasoning. For the task of multi-turn dialogue generation in open domain, several approaches^13–18^ for modeling hierarchy of the conversation using various frameworks and diversity-promoting objectives were proposed. Zhang et al.^19^ proposed a memory encoder which is trained with textual features to obtain dialogue representation, a decoder which is composed of an Recurrent Neural Networks (RNN) and a rule-memory network for response generation. Bidirectional Encoder Representations from Transformers (BERT)^20^ with around 340 million parameters successfully tackle a broad set of language understanding tasks. Denoising Sequence-to-Sequence Pre-training for Natural Language Generation, Translation, and Comprehension i.e BART^21^ with a bidirectional encoder^20^ and autoregressive decoder^22^ showed competitive performance on sequence classification as well as generation tasks. With 11 billion parameters, the T5 model^23^ is suggested to improve the performance for tasks requiring both natural language processing and generation. Generative Pre-trained Transformer (GPT)-3^24^ having 175 billion parameters obtains exceptional performance on a variety of tasks in the few-shot and zero-shot settings. Various pre-trained language models^1–3^ have demonstrated compelling performance on generating responses for dialogue systems by leveraging the pre-train / fine-tune paradigms. MASS^25^ jointly trains the encoder which takes as input a sentence with a randomly masked fragment (several consecutive tokens) and decoder that attempts to predict this masked fragment for text generation.

Several approaches integrating commonsense knowledge graphs with dialogue systems were demonstrated in^26–31^. Zhou et al.^27^ incorporated commonsense knowledge using static and dynamic attention to generate a correct response for a given post. Wu et al.^32^ proposed ConKADI which improved the selection and effective integration of facts which were incredibly pertinent to the context of the generated response. Young et al.^26^ augmented the large scale commonsense knowledge and integrated it with an end-to-end neural dialogue model in the form of external memory. Wu et al.^28^ proposed a model which integrates both knowledge graph and topic specific knowledge to improve the existing post-response pair generation models using a teacher-student recommendation network. Liu et al.^33^ proposed to predict entities in a structured way by encoding knowledge triplets using a neural knowledge diffusion module. Similarly, for an open-domain dialogue system, Liu et al.^34^ integrated the knowledge graph with the dialogue pipeline using three modules *viz.* knowledge augmentation to augment a knowledge graph with texts, knowledge selector and knowledge aware response generator to perform graph reasoning. A knowledge infused model for capturing semantic relations and to model conversation structures was proposed by Varshney et al.^35^ using a multi-hop attention mechanism.

Using large scale pre-trained language model, BART^36^ incorporated knowledge graphs using multi-head graph attention for commonsense reasoning. There are also findings Peters et al.^37^, He et al.^38^ that focused on inserting prior knowledge by using entity and relation embedding into deep neural language models. In order to train the models to distinguish between the right entity mention and randomly selected ones, WKLM^39^ replaced entity mentions in the original texts with names of additional entities of the same type. To align global information and language representation into the same semantic space, KEPLER^40^ optimised the models with mask language model objective and extracts knowledge from text by encoding the entities from their corresponding descriptions. A word-knowledge graph was used by CoLAKE^41^ to combine the language context and the knowledge context, and the extended mask language model aim was used to jointly learn a context aware representation for both language and knowledge. Sun et al.^42^ proposed knowledge-aware pre-training tasks to incorporate knowledge graphs into language models for language understanding and generation. To forecast the relation in the triple, the model must recognise mentions of both the head and tail entities in the associated sentence and establish the semantic link that exists between them. In relation extraction tasks, the essence of this procedure is identical to the distant supervision algorithm. Our work, on the other hand, only masks the head entities and predicts the tails entities that correlate to them, rather than the head entities themselves. This is done so that the model can learn to use the graph attributes in dialogues as well as the contextual information.

As the number of biomedical documents grows, so does the importance of biomedical text mining. With advancements in natural language processing (NLP), researchers have become more interested in extracting valuable information from biomedical literature, and deep learning has aided in the development of effective biomedical text mining models. Domain-specific language representation model such as Bidirectional Encoder Representations from Transformers for Biomedical Text Mining (BioBERT)^43^ and ClinicalBERT^44^ are pretrained utilising large scale data from biomedical domains and have demonstrated high quality performance on various medical tasks. They outperform significantly on three representative biomedical text mining tasks: biomedical named entity recognition, biomedical relation extraction, and biomedical question answering. Rao et al.^45^ developed a system that integrates language models with knowledge graph embeddings to provide understandable answers to queries from biologists. Auti et al.^46^ used the GAN-BERT architecture to categorize pharmaceutical texts, replacing the BERT model with the BioBERT model to capture domain-specific information. Yang et al.^47^ introduced the Pathway2Text dataset, which consists of 2,367 pairs of biomedical pathways and corresponding textual descriptions, and proposed a graph-based text generation method called kNN-Graph2Text that uses descriptions of related graphs to generate new descriptions. Luo et al.^48^ developed the BioGPT language model, a domain-specific generative Transformer that was trained on a large volume of biological literature and tested on six biomedical natural language processing tasks. Blanc et al.^49^ used FlauBERT and CamemBERT to design a study to ascertain which language model and neural network architecture combination was best for intent and slot prediction by a chatbot using a French corpora of clinical cases.

Zeng et al.^50^ released a high-quality medical dialogue dataset in Chinese and English that covers more than 50 diseases. There are 3.4 million patient-doctor dialogues, 11.3 million utterances, and 660.2 million tokens in the Chinese dataset, while there are 0.26 million conversations, 0.51 million utterances, and 44.53 million tokens in the English dataset, which covers 96 illnesses categories. Liu et al.^51^ released another high-quality chinese medical dialogue dataset containing 12 types of common Gastrointestinal diseases named *MedDG*, with more than 17K conversations. They put forth two challenges, the first of which is to predict the subsequent entities based on dialogue contexts, and the second of which is to generate virtual doctor’s responses. On four pediatric disorders namely upper respiratory infection, functional dyspepsia, infantile diarrhea, bronchitis, the CMDD dataset^52^ containing 2,067 dialogues was released. This dataset did not handle the problem of data imbalance between diseases. A much larger medical dialogue dataset, namely Chunyu^53^ was proposed to improve upon the previous model, which now contains 15 diseases with comparatively distinct data ratios.

In the medical domain, on the MedDialog(CN) dataset, an end-to-end variational bayesian generative strategy^9^ was developed to generate medical dialogue by approximating posterior distributions over patient states and physician actions. In order to test the performance of their proposed *VRBot* model, they curate a large medical dialogue dataset with over 60,000 medical conversations having 5,682 entities (such as Asthma and Atropine). Liu et al.^54^ proposed an effective technique by auto encoding knowledge graphs for multimodal medical report generation using a knowledge-driven encoder and a knowledge-driven decoder. Similarly, Liang et al.^55^ presented a lightweight as well as a scalable mechanism using the transformer and BERT-GPT architecture to integrate the medical knowledge into different neural generative models on the MedDG and the MedDialog(CN) dialogue corpora. Lin et al.^53^ proposed a low-resource medical dialogue-generating system along with a Graph-Evolving Meta-Learning (GEML) framework that learns to evolve the commonsense graph for reasoning disease-symptom connections.

In this work, we exploit the largest conversational medical corpus, MedDialog(EN)^50^ containing 0.26 million English consultations between the patients and doctors, and demonstrate how external medical knowledge can improve the task of medical dialogue generation. We propose a knowledge-aware neural medical conversation model named *MED* to generate contextualized entity embeddings for incorporating relevant information from the knowledge graphs in accordance with the diverse conversation structures. Furthermore, we annotate the utterances with symptoms, diseases, tests, and other pertinent categories using semi-automated NLP techniques which are further used to enhance the knowledge graph retrieved using UMLS^10^ database. The efficacy of our proposed approach is demonstrated through our experiments on two medical dialogue corpus *viz.* MedDialog(EN) and Covid Dataset, a newly augmented and much larger corpora focusing mainly on Covid-19. Our method allows the use of a large-scale biomedical knowledge graph to facilitate the understanding of the clinical entities and relations present in the current dialogue and the generation of a response with clinically correct information.

## Methodology

**Problem definition:** The objective of this research is to build a virtual doctor (i.e. conversational agent), which could assist the users (i.e. patients and/or other stakeholders) by providing an appropriate response. Given the current utterance and contextual conversations between patients and doctors, represented as a sequence of words, the task is to generate medically relevant responses. To generate more knowledgeable and engaging dialogue, we make our conversational agent knowledge grounded by leveraging an external knowledge graph.

**Conversation:** We have a set of conversations, each consisting of multiple turns, where the patient and the doctor take turns speaking. For each turn, we have the patient’s utterance and the doctor’s response, both represented as a sequence of words.

- Consider a conversation $C = \{c_i\}_{i=1}^K$ consisting of *K* turns, where each turn is represented by a tuple $(b_k, d_k)$. $b_k$ denotes the patient’s utterance and $d_k$ is the virtual doctor’s response at the *k*-th turn.
- The patient’s utterance $b_k$ is a sequence of $\left( b_{k,1}, b_{k,2}, \ldots , b_{k,|b_k|}\right)$ words, where $|b_k|$ denotes the total number of words in the utterance. Similarly, the doctor’s response $d_k = \left( d_{k,1}, d_{k,2}, \ldots , d_{k,|d_k|}\right)$, comprising of $|d_k|$ words.

**Knowledge Graph:** The augmented medical knowledge graph is denoted as: *G* = $\left( g_{1},g_{2},\ldots ,g_{|b_k|}\right)$.

- G is a set of graphs, each graph is a set of triples, and each triple is a tuple of head, relation, and tail entities.
- Each graph, $g_i$, contains a set of triples $g_i = \left( {\tau }_1, {\tau }_2, \ldots , {\tau }_{|g_i|}\right)$.
- Each triple ${\tau }_j$ has $({\textbf {h}},{\textbf {r}},{\textbf {t}})$ denoting head, relation, and tail entity respectively, with ${|g_i|}$ indicating the total number of triples for every graph $g_i$.

We create the augmented medical knowledge graph in the following ways:

1. We first take as input an utterance, $b_k$, and initialize the first word as the first node for the graph, $g_i$. For every node, we extract different subgraphs using UMLS (section “Construction of the medical knowledge graph using UMLS”). Starting from the first node in $g_i$, the agent follows a path in the graph and stops at a node that it finds in the doctor’s response.
2. We extract medical entities from each utterance and use the four different annotated entity categories for defining relationships between them (section “Construction of the medical entity graph”). We add these new triples to the subgraphs extracted from UMLS.

### Medical knowledge graph

**Figure 1:**
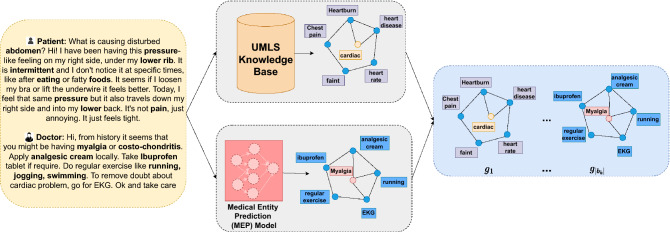
Medical Knowledge Graph: The patient-doctor dialogue as shown on the left is first passed through the *Quick-UMLS tool*, which is used to extract the medical concepts and relations from the UMLS knowledge base as shown in the box on top-middle. The box on the bottom-middle uses the MEP model to discover additional biomedical words present in the dialogues. Finally, the augmented knowledge graph used in the dialogue generation pipeline is shown in the right.

Utilizing the knowledge graph is driven by the fact that generating responses for the virtual doctor typically necessitates specialized medical knowledge. As a result, we can use the knowledge graph, which simulates the knowledge structure specific to the medical domain. Specifically, we build a global medical knowledge graph $g_i = ({\tau }_1, {\tau }_2, \ldots , {\tau }_{|g_i|})$ where each triple covers the most common diseases and symptoms. In this section, we describe the steps to form a large augmented graph of disease-symptom relationships using medical concepts extracted from several sources. The process for preparing the medical knowledge graph is shown in Fig. 1 and explained in sections “Construction of the medical knowledge graph using UMLS” and “Construction of the medical entity graph”.

#### Construction of the medical knowledge graph using UMLS

The UMLS^10^ is a vast knowledge base that contains huge number of concept names and relationships between various health and biomedical words. The three knowledge sources available in UMLS are Metathesaurus, Semantic Network, and Lexical Tools. We use the Metathesaurus and the Semantic Network modules to implement our approach. Metathesaurus contains over 1 million biomedical concepts, and each concept is related to the other concept in some way. Furthermore, each concept is assigned with one or more semantic types, and their relationship with the semantic types is determined using a semantic relationship network. The semantic network contains 127 semantic types and 54 relationships between them. We use the QuickUMLS^56^ (https://github.com/Georgetown-IR-Lab/QuickUMLS) tool to extract the required medical concepts and construct the clinical graphs.

Quick-UMLS is used to extract biomedical entities from the UMLS Metathesaurus and associate them with unique identifiers (CUIs) and semantic type. It takes the utterances from the medical conversation as input, finds the closest match in the UMLS set of strings, and then returns the CUI, or list of semantic categories, for each concept in the utterance. Each unique identifier (CUI) in UMLS is treated as a node in our knowledge graph, and then using the semantic network module from UMLS appropriate relationship between each node is determined. Let us say, we have an input utterance as “Hello doctor, how to improve fetal growth and heart beat ?”. We utilise the Semantic Network to create a sub graph for the medical concept “heart beat” and form the following set of triples: (’heart beat’, ’*affects*’,’anxiety’),(’heart beat’, ’*affects*’, ’circulation’), (’heart beat’, ’*affects*’, ’collapsed lung’), (’heart beat’, ’*process of*’, ’unconscious’) and so on. This is shown in the top side of the Fig. 1.

The UMLS knowledge base overlooks some important medical information found in many medical dialogue datasets, such as medical tests, medication, and home remedies even after using all the 54 relationships and 127 semantic types from the UMLS semantic network. We use a neural BERT based medical entity prediction model to annotate the dialogues with symptoms, medical tests, medications, and home remedies in order to enrich the knowledge graph obtained from UMLS.

#### Construction of the medical entity graph

**Medical Entity Annotation:** We choose the following four different kinds of entities for annotation after consulting with domain experts: *Symptom* such as chest pain, thyroid dysfunction and so on; *Medication* such as analgesic, pantoprazole and so on; *Home Remedy* such as regular exercise, jogging, and so on; and *Medical Test* such as x-rays, blood tests and so on. The utterances of the conversation are labeled individually with four categories of entities using Named Entity Recognition (NER) method as the annotation technique, as illustrated in Table 3.

Four annotators with relevant backgrounds participated in the annotation process. The annotators have a mix of qualifications that includes linguistics with PhDs, medical experts with medical degrees, specifically masters in medicine. This combination of qualifications ensures that the annotators have the necessary linguistic skills, as well as the medical expertise to accurately understand and interpret the medical texts. The annotators are regular employees who are paid at a rate of $35,000 per month (in accordance with university standards). The annotators have been working on related projects for the past three years as members of our research team. They talk about making an annotation template first. Four blank columns are offered to the annotators so they can select the appropriate medical phrase for the various categories. For example in Table 3, for the first utterance, the relevant medical entities to be annotated are *Symptom*: Myalgia, costo-chondritis; *Medication*: analgesic cream, Ibuprofen; and so on. We present a few triples from the UMLS knowledge base, along with the corresponding utterances in the first column, to demonstrate the difference in the information obtained using different methods of graph creation. The triples are shown in the last column. Each participant annotates a small portion of the data and reports the confusing utterance. They summarize their observations and then revise the annotations once more.

To ensure that the guidelines are effective, a number of pilot studies were conducted with linguistics on around 200 test dialog samples. These pilot studies were used to test the annotation guidelines and identify any inconsistencies or issues that needed to be addressed. After the pilot studies, the annotation guidelines were discussed with medical experts, who verified the annotations. Any inconsistencies found in the pilot studies were removed and resolved in order to ensure consistency in the annotation process. This process guarantees that the final annotation guidelines are accurate and reliable, which is essential for the credibility of the research. Inconsistencies were errors, ambiguities, and sometimes a lack of clarity in the annotation guidelines. They were resolved by revising the guidelines or providing additional training for the annotators. Additionally, we took care to clearly define the concepts and entities that needed to be annotated.

We observe a Fleiss’ kappa^57^ score of 0.89 between annotators denoting great agreement between them for the entity annotation task. However, in addition to Fleiss kappa, we compute Krippendorff’s Alpha^58^: a measure of inter-rater variability. We obtain Krippendorff’s Alpha score of 0.62 for medical entity annotation indicating a moderate inner-annotator agreement. We annotate the first 6K utterances using the above method from the Covid Dataset and then using the algorithm described in the following paragraph, we annotate both the MedDialog Dataset and remaining conversation in the Covid Dataset.

**Medical Entity Prediction (MEP) Model:** To recognise medical entities from the dialogue context *C*, we use the MEP model described as follows. We first annotated the 6K samples manually tagged with entities as described previously using the Inside-Outside-Beginning (IOB)-tagging as the annotation scheme. Each tag indicates whether the corresponding token is inside, outside, or at the beginning of a specific named entity. We had four labels: Symptom, Medication, Medical test, and Remedy, for a total of nine tags: B-Medication, I-Medication, B-Symptom, I-Symptom, B-Medical test, I-Medical test, B-Remedy, I-Remedy, B-remedy, I-remedy, and O. We then fine-tune the pre-trained BERT base model (Uncased: hidden-768, heads-12, layer-12) for the medical entity prediction task. The input is in the form of a tokenized sentence, and the tokens are truncated or padded according to the maximum length of the model. The training was done on 80% of the total data (4978 sentences) while validation was done on the remaining 20% (1244 sentences). For evaluation, we calculate the precision, recall and F1 score for each label using seqeval.metrics (https://pypi.org/project/seqeval/). The scores from all the metrics are shown in Table 2 and the predictions from the *MEP* model are shown in Table  3. Using the trained model, we annotate all the utterances of the MedDialog Dataset and Covid Dataset. Finally, the medical entity graph is built with the extracted medical entities as nodes and edges for each pair of medical entities that appear in the four different categories.

**Table 2:** Precision, Recall and F1-scores for the MEP Model.

Labels	Precision	Recall	F1-score %
Medication	0.70	0.76	73.1
Symptom	0.73	0.81	77.2
Medical Test	0.76	0.77	76.1
Home Remedy	0.44	0.65	53.2
Micro Avg	0.70	0.79	74.0
Macro Avg	0.66	0.75	70.0
Weighted Avg	0.71	0.79	74.4

**Table 3:** Predicted entities using MEP model.

Utterance	Symptom	Medication	Medical tests	Home remedies	Triples from UMLS
Hi, From history it seems that you might be having Myalgia or costo- chondritis. Apply analgesic cream locally. Take Ibuprofen tablet if require. Do regular exercise like running, jogging, swimming . To remove doubt about cardiac problem, go for EKG. Ok and take care.	Myalgia, costo-chondritis	analgesic cream, Ibuprofen	EKG	regular exercise, running, jogging, swimming	(cardiac, part_of, probiotics), (analgesic, diagnoses, chronic_infection), (tablet, conceptual_part_of, surgery), (jogging, isa, relaxation)
Hello! Thank you for asking on HCM! Regarding your complains, I recommend to consult your doctor (internist or cardiologist) for a medical check up (physical exam, resting ECG and some blood tests) to exclude a heart rhythm dysfunction, anemia, thyroid dysfunction, etc , If a heart rhythm disorder is suspected, an ambulatory ECG monitoring is advisable. Metoprolol dose and titration scheme should be reconfirmed. Hope to have been helpful. Greetings!.	heart rhythm dysfunction, anemia, thyroid dysfunction	Metoprolol	ECG, blood tests, ECG	resting	(cardiologist, uses, eosinophils), (resting, location_of, hepatitis_b), (anemia, associated_with, difficulty_breathing), (thyroid, diagnoses, chronic_infection)
Seems to be muscle ache. Take a 7 day rest. Take any muscle relaxant like tizanidine, hot compress and local analgesic ointment. Do a chest x ray pa view to exclude any lung disorder and be sure the pain is not due to any heart cause. Complete a course of oral analgesic for 7 days with a PPI like pantoprazole.	muscle, pain, heart cause	tizanidine, analgesic, pantoprazole	chest X-ray	rest, hot compress, ointment	(muscle ache, manifestation_of, benzodiazepines), (disorder, associated_with, difficulty_breathing), (heart, degree_of, pulmonary_eosinophilia), (x ray, measures, acute_cystitis)
Hey there, Looking to your case its chronic pain in chest which may be from chest wall or lungs , as your EKG and TMT is normal. Go for chest X-ray, rule out cause of lung disease. Else take analgesic and hot fomentation. may be from chest wall or lungs, as your EKG and TMT is normal. Go for chest X-ray, rule out cause of lung disease. Else take analgesic and hot fomentation.	chest pain, lung disease	analgesic	EKG, TMT, chest X-ray	take analgesic hot fomentation	(chronic pain, evaluation_of, punctate_keratitis), (lung disease, associated_with, difficulty_breathing), (chest wall, location_of, hepatitis_b), (chest, location_of, both_legs)

For example, for the input sequence *“Hi, from history it seems that you might be having myalgia or costo-chondritis. Apply analgesic cream locally. Take Ibuprofen tablet if require. Do regular exercise like running, jogging, swimming. To remove doubt about cardiac problem, go for EKG. Ok and take care”* medical concepts such as *analgesic, tablet, exercise, swimming, cardiac, care and so on* and triples such as *(analgesic, diagnoses, heart diseases), (cardiac, location of, heart sounds), (swimming, isa, relaxation) and so on* are extracted from the UMLS database. As can be clearly seen, UMLS fails to incorporate medical concepts such as myalgia, costo-chondritis and ibuprofen. We make an attempt to extract these concepts using our MEP model. Examples of the extracted medical concepts can be seen in the Table 3. Using the extracted medical concepts from entity prediction model, we form an entity graph having the following nodes and relations: *(Myalgia, medication, analgesic cream), (Myalgia, medication, ibuprofen), (Myalgia, medical test, EKG), (Myalgia, medication, ibuprofen), (Myalgia, remedies, regular exercise), and so on*. This is finally appended with the triples obtained from the UMLS database to form the new augmented medical graph and finally used in section “Model description” for building knowledge-aware medical dialogue systems.

### Model description

**Figure 2:**
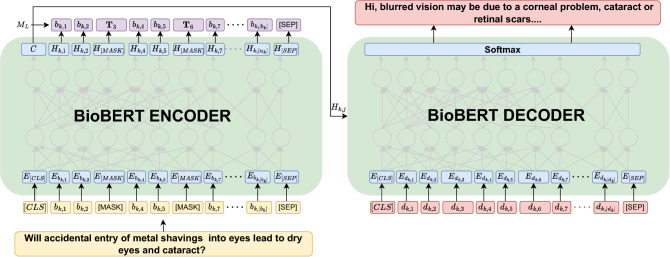
MED architecture: The proposed architecture first encodes the conversation history using a BioBERT encoder as shown in the left. We first attempt to predict the sequence $M_L$ using the token classification task that provides the hidden states, $H_{k,i}$, for the input sequence. This input sequence is then fed into a BioBERT decoder to generate the response.

In this part, we outline our suggested method for having the virtual doctor produce responses to patient inquiries that are appropriate from a medical standpoint. Figure 2 depicts the detailed model architecture proposed for the task of medical dialogue generation. We use the BioBERT BASE^43^ as the pre-trained language model to dynamically build contextualised representations for the input sequences using graphical knowledge. With the conversation sequence *C* in hand, we first mask the tokens for which we acquire a medical graph $g_i$ in accordance with section “Construction of the medical entity graph”, and instead of predicting the token itself, we infer the tail entities extracted from the corresponding graphs. We call this as the MED model. For example, given the triples: ’(cough, *treats*, eosinophilia)’, ’(blockage, *occurs in*, pulmonologist)’, we mask the tokens *cough* and *blockage* in the dialogue context, as *“i am continuously suffering from [MASK] due to feeling of some [MASK] in upper wind pipe”*; the model then predicts the tail entity corresponding to the masked tokens *cough* and *blockage* i.e *eosinophilia* and *pulmonologist*. We choose the tail token from the first triple in the sequence $({\tau }_1, {\tau }_2, \ldots , {\tau }_{|g_i|})$, associated with the m-th masked token and denote it as ${\textbf {T}}_m$. Given the input sequence, *I* (Each token in *I* is first passed through a series of three embedding layers (Token, Segment, and Position). The resulting embeddings are concatenated and denoted as $E_{b_{k,i}}$), we first attempt to predict $M_L$ by using BERT based token classification model which returns the hidden states, $H_{k,i}$, for the input sequence which is usually the representation of the [*CLS*] token. While predicting any masked token, information from nearby words is utilized. Our approach directly utilizes the dialogue context, (*I*) , simultaneously incorporating the knowledge triples.

We first form the flattened token sequence for the input utterance with the masked entities:

1 $$\begin{aligned} I= & {} {[CLS], b_{k-1,1}, [MASK], b_{k-1,3}, \ldots , b_{k-1,|b_k-1|}, [SEP], d_{k-1,1}, [MASK], \ldots ,d_{k-1,|d_{k-1}|}}, \nonumber \\&\quad {[SEP], b_{k,1}, b_{k,2}, [MASK], b_{k,4}, b_{k,5}, [MASK], b_{k,7}, \ldots , b_{k,|b_k|}, [SEP]} \end{aligned}$$

The corresponding ground truth label, $M_L$, for token classification is shown as follows:

2 $$\begin{aligned} M_L= & {} {[CLS], b_{k-1,1}, {\textbf {T}}_{1}, b_{k-1,3}, \ldots , b_{k-1,|b_k-1|}, [SEP], d_{k-1,1}, {\textbf {T}}_{2},\ldots , d_{k-1,|d_{k-1}|}, [SEP], b_{k,1}, b_{k,2},{\textbf {T}}_3 , b_{k,4}, b_{k,5},} \nonumber \\&\quad {{\textbf {T}}_6 , b_{k,7}, \ldots , b_{k,|b_k|}, [SEP]} \end{aligned}$$

where the [*CLS*] token is inserted at the beginning of the sequence as an indicator of the start of the sentence. The [*SEP*] token distinguishes one sequence from the next and indicates the end of a sentence.

The BioBERT-based decoder generates text by predicting one word at a time, using the hidden state $H_{k,i}$ at each time step. During training, the decoder is provided with the actual next word in the sequence, taken from the set $R_L$, as input. However, during inference, the decoder uses the word it has previously predicted as input. To start the decoding process, the first input to the decoder is taken from the first token in $R_L$.

3 $$\begin{aligned} R_L = [CLS], d_{k,1}, d_{k,2}, d_{k,3}, d_{k,4}, d_{k,5}, d_{k,6}, d_{k,7},\ldots , d_{k,|d_k|}, [SEP] \end{aligned}$$

The BioBERT model uses its hidden state from the top layer, passed through a linear layer, to predict the next token in the target (output) sequence.

4 $$\begin{aligned} P(y_{k,j}) = \text {softmax}\left( {W}_{1}[H_{k,j}] + b_1 \right) \end{aligned}$$

where $W_1$ is a learnable weight matrix and $b_1$ is the bias.

The decoder loss is the cross-entropy between the output distribution $P(y_{k,j})$ and the reference distribution, $d_j$, denoted as $Loss = - {\sum }{d_j}log(P(y_{k,j}))$.

### Ethical declaration

The public can access all of the datasets used in this study freely. By crawling additional data from the sources cited in the datasets’ respective papers, we also add to the dataset new patient-doctor conversations. A committed group of full-time employees were employed for annotation purposes. All experiments were conducted in conformity with the applicable rules and guidelines. Medical dialogue systems might be employed in the real world to give patients advice and gather information for diagnoses. Doctors will eventually take control of the procedure, even if the agent makes a few minor errors along the way.

## Datasets and experimental setup

In this section, we describe the datasets that we use for our experiments, implementation details, baseline and the evaluation metrics. The train, test and validation split details for both the *MedDialog* and *Covid Dataset* are mentioned in Table 4. More details of the dataset containing information on the number of utterances and so forth are mentioned in Table 5.

### MedDialog(EN) dataset

The MedDialog(EN)^50^ dataset is the largest available dataset which comprises of 0.26 million patient-doctor conversations. Each conversation is composed of two sections: a comprehensive description of the patient’s medical issue, including symptoms, duration of the illness and other relevant information, as well as the physician’s recommended course of treatment and suggested remedies. The dataset includes 96 distinct medical specialities, such as andrology, cardiology, nephrology, and pharmacology, as well as 51 different categories of illnesses, such as diabetes, geriatric concerns and pain management. The total number of words in the corpora is 32,855,023, and the total number of utterances is 396,562. The average, maximum, and minimum number of tokens in an utterance are 83.0, 3,677, and 1, respectively. The average, maximum, and minimum number of utterances are 2.0, 2.0, and 2.0, respectively. Sample conversation from the dataset is shown in Table 1.

### Covid dataset

We enhance the CovidDialog dataset^59^ having 603 conversations with the diseases which are the symptoms of Covid-19 such as fever, cough, allergies, and so forth. The reason for this is because dialogues regarding fever, cough, cold, and other Covid-19 symptoms can be helpful in response generation on the Covid-19 disease. The dataset now includes over 10,000 English conversations about Covid-19 and symptoms associated with Covid, which helps in the creation of a resourceful medical dialogue system. Each session starts with a brief overview of the patient’s health issues, which is followed by a chat between the patient and the doctor. The total number of utterances and tokens in the dataset is 26,658 and 2,735,112, respectively. The average, maximum, and minimum number of utterances are 3.0, 42, and 2, respectively. The average, maximum, and minimum number of tokens in an utterance are 103.0, 1509, and 1, respectively.

**Table 4:** Dataset details.

MedDialog				Covid dataset		
	Train	Valid	Test	Train	Valid	Test
#Conversation	169,530	21,192	21,193	7,482	936	964
#Utterances	317,244	39,656	39,662	21,500	2,538	2,620

**Table 5:** Dataset statistics.

Statistics	MedDialog			Covid dataset		
#Utterances	396,562			26,658		
#Tokens	32,855,023			2,735,112		
Average # Utterances	2			3		
Maximum # Utterances	2			42		
Minimum # Utterances	2			2		
Average # Tokens	83			103		
Maximum # Tokens	3,677			1,509		
Minimum # Tokens	1			1		

## Implementation details

All the experiments are implemented using Pytorch. We chose the hidden size of 512 for all our model. The number of layers is set to 2 for BERT and BART based models, and 6 for the BioBERT and MED model. We use the cased models for every pre-trained model. We use the ADAM optimizer whose learning rate is fixed to 0.0005 and set the beam size to 1, while decoding the responses. The input utterances are truncated to a max token count of 400 and output utterances to 100. We choose the best model when the loss on the validation set does not decrease. We use the GeForce GTX 1080 Ti as the computing infrastructure. Each model is trained up to 30 epochs.

### Baselines

We use the following baseline models: **CCM**^27^ and **ConKADI**^32^ for incorporating knowledge graphs for dialogue generation. Pre-trained language models, such as ${\textbf {DialogGPT}}_{{{\varvec{finetune}}}}$^2^, **BERT**^20^, **BART**^21^ and **BioBERT**^43^ for dialogue generation.

1. **CCM**^27^: Many tasks involving natural language processing require commonsense knowledge. To illustrate how extensive commonsense knowledge can aid language interpretation and generation, this model first forms an appropriate knowledge graph from a knowledge base. The graph is then encoded using a static graph attention technique, which improves the post’s semantic content and makes it easier to understand. The model then reads the extracted knowledge graphs and knowledge triples while generating each word of the response using a dynamic graph attention strategy within each graph and thus aids in improving the whole dialogue generation process. We replicated the results of *CCM* on our dataset using the codes in this link : https://github.com/thu-coai/ccm.
2. **ConKADI**^32^: To make the dialogue models aware of the context when using the knowledge, ConKADI provides a Felicitous Fact mechanism to help the model focus on knowledge facts that are extremely relevant to the context in order to help the dialogue models be aware of the context when integerating the knowledge. Context-Knowledge Fusion and Flexible Mode Fusion are two further strategies that aid in combining the knowledge information for the model. Additionally, ConKADI employs trainee responses as posterior knowledge. The codes for generating the results on the dataset can be found in this link: https://github.com/pku-sixing/ACL2020-ConKADI
3. ${\textbf {DialogGPT}}_{{{\varvec{finetune}}}}$^2^: Dialogue generative pre-trained transformer (DialogGPT), a large scale fine-tuned neural conversational response generation model, was constructed using the OpenAI GPT-2 architecture and trained with 147 million Reddit conversations. Additionally, the model that performed the best was selected with 345M parameters. The GPT-2 transformer model uses a stack of masked multi-head self attention layers and the generic transformer language model to train on the large amount of web-text data. The codes for replicating the results can be found in the link as follows: https://github.com/UCSD-AI4H/COVID-Dialogue/tree/master/src/DialogGPT/GPT2-chitchat-master
4. **BERT**^20^: Transformer, which makes use of an attention mechanism to learn contextual relationships between words (or, sub-words) in a text, is used by BERT. They simply mask a portion of the input tokens at random, then predict those masked tokens in order to train a deep bidirectional representation. Using the BERT model as both an encoder and a decoder, the input utterances from the conversations are encoded, and the pertinent output is generated. Codes to obtain results is possible using the codes in this link: https://github.com/UCSD-AI4H/COVID-Dialogue/tree/master/src/Transformer
5. **BART**^21^: BART comprises of a denoising autoencoder utilized for pre-training sequence-to-sequence models. BART is trained by altering documents and then optimizing the cross-entropy loss between the original document and the decoder’s output. It uses a sequence-to-sequence approach for emcoding the corrupted text with a bidirectional encoder and a autoregressive decoder works from left to right. For pre-training purposes, the negative log likelihood of the original document was optimized. For our datasets, the sentences in the conversations are encoded using the bidirectional encoder, and the relevant response is generated using autoregressive decoding. The codes for implementing the *BART* model is available in the link: https://github.com/UCSD-AI4H/COVID-Dialogue/tree/master/src/Bert-GPT
6. **BioBERT**^43^: The BioBERT model adopts the previously described *BERT* architecture except that it has been pre-trained on a large-scale biomedical corpora (PMC full-text articles and PubMed abstracts). In a number of biomedical text mining tasks (NER, RE and QA), it has shown to outperform BERT and other state-of-the-art models. In order to adapt the *BioBERT* model, we encode the dialogue history using the BioBERT model as an encoder. Finally, the decoding process is completed by utilizing a BioBERT model as a decoder. Codes to replicate this model can be found on the link:  https://github.com/deekshaVarshney/MED

## Evaluation metrics

**Automatic evaluation:** We evaluate our models on the test set of both the MedDialog(EN) and Covid Dataset, using the following standard metrics. We use one of the most popular metrics for evaluating sequence like BLEU^60^, F1-score, Perplexity (PPL)^61^ and n-gram diversity (Div.)^62^ to automatically evaluate the quality of generated responses.

1. **Perplexity:** We make use of perplexity as one of the automatic evaluation metric for the medical dialogue generation task. It is a measurement of how well a model can predict human responses. Generally, lower perplexity indicates better generation performance. It is defined in Equation 5. Our various models are tested on the generation ability by computing perplexity on the test data.
  5 $$\begin{aligned} PPL = \text {exp} \{{\frac{-1}{N} \sum _{i=1}^{N} log(p(y|U))}\} \end{aligned}$$
  where *N* is the total number of samples in the test set.
2. **BLEU (Bilingual Evaluation Understudy Score):** We measure the accuracy of the generated responses by using BLEU, a word-based metric which measures n-gram overlaps with the gold response. First, sentence by sentence n-gram matches are calculated. Then the total number of candidate n-grams in the test corpus are multiplied by the sum of all the clipped n-gram counts for the candidate sentences to arrive at a modified precision score, for the full test corpus. The BLEU metric is in the range of 0 to 1. Unless they are exact replicas of a reference sentence, few sentencess will receive a score of 1.
3. **F1-score** (https://github.com/facebookresearch/ParlAI/blob/master/parlai/core/metrics.py): We also compute *unigram* F1-score between the predicted sentences and the ground truth sentences. We first compute the precision using the number of overlapping unigram words between the gold and the generated sentence divided by the total number of words in the gold response. Second, we compute the recall using the count of unigram overlap divided by the count of words in generated text. Finally, the F1 score is computed as defined below:
  6 $$\begin{aligned} F1 = \frac{2 \cdot recall \cdot precision}{recall + precision} \end{aligned}$$

Embedding-based metrics^63^, such as Greedy Matching, Vector Extrema and Embedding Average are an alternative to word-matching-based metrics. Code to implement the above metrics: https://github.com/Maluuba/nlg-eval. They are defined as follows:

1. **Greedy matching**: Given two sequences *q* and ${\hat{q}}$, the total score is calculated by greedily matching each token *w* $\in$ *q* with a token ${\hat{w}}$ $\in$ ${\hat{q}}$ based on their word embeddings’($e_w$) cosine similarity and finally averaged across all words. It is mathematically defined as follows:
  7 $$\begin{aligned} G(q, {\hat{q}}) = \frac{\sum _{w \in q;} max_{{\hat{w}} \in {\hat{q}}} cos\_sim(e_w, e_{{\hat{w}}})}{|q|} \end{aligned}$$
  Due to the asymmetry of this algorithm, we must average the greedy matching scores *G* in both directions.
  8 $$\begin{aligned} GM(q, {\hat{q}}) = \frac{G(q, {\hat{q}}) + G({\hat{q}},q)}{2} \end{aligned}$$
2. **Embedding Average**: The mean of the word embeddings of each token in a sentence *q* is known as the embedding average ($\bar{e}$) or the sentence embeddings.
  9 $$\begin{aligned} \bar{e}_q = \frac{\sum _{w \in q}e_w}{|\sum _{w' \in q}e_{w'}|} \end{aligned}$$
  Then the cosine similarity between two sentence level embeddings, one representing the ground truth response *q* and the other representing the retrieved / generated response ${\hat{q}}$ is calculated as described below:
  10 $$\begin{aligned} EA = cos(\bar{e}_q, \bar{e}_{{\hat{q}}}). \end{aligned}$$
3. **Vector Extrema**: For each dimension of the word vectors, take the most extreme value amongst all word vectors in the sentence, and use that value in obtaining the sentence-level embedding:
  11 $$\begin{aligned} e_{qd} = {\left\{ \begin{array}{ll} max_{w \in q} e_{wd} &{} \text {if }e_{wd} > |min_{w' \in r} e_{w'd}| \\ min_{w \in q} e_{wd} &{} \text {otherwise} \end{array}\right. } \end{aligned}$$
  where *d* denotes a vector’s dimensions and $e_{wd}$ denotes the embedding at the $d-th$ dimension. We again compute the cosine similarity between the sentence-level vectors to obtain VE.

**Human evaluation:** To evaluate the quality of generated responses from a human perspective, we randomly select 50 dialogues from each model developed using the MedDialog and Covid datasets and analyse the predicted responses with the assistance of three human evaluators. Human raters are post-graduates in science and linguistics with annotation experience for text mining tasks especially in the medical domain. The annotators are regular employees and receive a monthly salary of approximately $35,000. (in accordance with university standards). As a part of our research team, the annotators have been working on related projects for the previous three years. We also had a physician with a postgraduate degree in medicine validate our model’s results. The responses were confirmed to have maintained the crucial medical information. To assess the accuracy of our model predictions, we employ the following metrics:

1. Fluency: This is a measure of sentence grammatical accuracy (i.e no fragments or missing parts) and that the text is easy to understand.
2. Adequacy: This metric is used to determine whether the generated response is meaningful and relevant to the conversation history.
3. Entity Relevance: This metric is used to determine whether or not a response contains the correct medical entities which are mentioned / referred in the conversation.

The scale runs from 1 to 5. The higher the number, the better. The ratings for the fluency metric are incoherent, disfluent, non-native, satisfactory, and perfect English, respectively. For the adequacy metric, these correspond to none, little meaning, much meaning, most meaning, and all meaning, respectively. Similarly, for Entity Relevance, we give the higher score if the response contains all the entities mentioned in the ground truth dialogue. As the final results, the ratings from the various annotators are averaged. To assess inter-annotator agreement, we compute the Fleiss’ kappa score^57^.

## Results and analysis

Tables 6 and 7 show the automatic and human evaluation results for the baselines and the proposed models.

### Automatic evaluation

The results of using automatic evaluation metrics on both the dataset are shown in Table 6. On most metrics, we see that *MED* outperforms the baseline models, demonstrating the efficiency of introducing global and data specific medical information generated using the MEP model for the task of medical dialogue generation. Our proposed model outperforms the knowledge graph based models, CCM^27^ and ConKADI^32^ on all the automatic evaluation metrics as well as the embedding-based metrics, demonstrating the importance of masking and predicting relevant medical concepts in generating relevant responses. When compared to the strongest baseline, BioBERT, *MED* yields a significant performance improvement of around 5.2 F1-score points and 8.3 BLEU-4 points on the test-set of the MedDialog corpora. On the Covid Dataset also, *MED* outperforms the BioBERT baseline on F1 and BLEU-4 evaluation metrics by 8.5 and 9.3 points, respectively.

**Table 6:** Automatic evaluation results for the baseline and suggested model were achieved using the MedDialog(EN) corpus and the Covid Dataset.

	MedDialog(EN)						Covid Dataset					
Models	PPL	F1%	BLEU-4	Embedding Average	Vector Extrema	Greedy Matching	PPL	**F1%**	BLEU-4	Embedding Average	Vector Extrema	Greedy matching
CCM	100.34	10.3	0.013	0.756	0.282	0.609	96.32	9.3	0.008	0.708	0.270	0.598
ConKADI	89.90	13.4	0.020	0.830	0.302	0.608	84.23	11.4	0.018	0.723	0.302	0.630
$\text {DialogGPT}_{{\textit{finetune}}}$	65.34	13.3	0.015	0.750	0.271	0.619	55.32	10.1	0.016	0.718	0.271	0.597
BERT	37.44	20.4	0.038	0.910	0.355	0.704	38.07	20.0	0.026	0.903	0.362	0.705
BART	22.417	20.5	0.047	0.907	0.357	0.698	23.954	19.1	0.025	0.906	0.358	0.696
BioBERT	21.474	20.8	0.048	0.908	0.361	0.700	22.355	20.0	0.032	0.906	0.357	0.700
**MED**	**20.622**	**21.9**	**0.052**	**0.921**	**0.373**	**0.725**	**20.512**	**21.7**	**0.035**	**0.919**	**0.369**	**0.728**
MED-UMLS	20.843	21.4	0.048	0.914	0.370	0.721	20.765	20.6	0.034	0.912	0.362	0.708
MED-MEP	21.702	18.9	0.042	0.911	0.370	0.719	22.513	19.7	0.031	0.903	0.359	0.713
MED-H	22.732	20.9	0.042	0.901	0.371	0.702	23.112	20.3	0.032	0.909	0.359	0.700

The bolded results represent the best outcome for the metric, with statistically significant improvement over the best baseline (t-test with p-value at 0.05 significance level).

**Table 7:** Human assessment results for the baseline and proposed model on the MedDialog(EN) and Covid dataset.

	MedDialog(EN)				Covid Dataset			
Models	Fluency	Adequacy	Entity Relevance	Kappa	Fluency	Adequacy	Entity Relevance	Kappa
BERT	2.08	1.72	1.65	0.84	1.58	1.46	1.42	0.84
BART	2.45	2.17	2.04	0.86	2.74	2.43	2.49	0.86
BioBERT	2.82	2.54	2.46	0.86	3.42	2.90	2.60	0.87
**MED**	**3.56**	**2.66**	**2.52**	**0.83**	**3.54**	**3.05**	**2.87**	0.85

The bolded values represent the best value for each statistic.

### Human evaluation results

Table 7 displays the results of human evaluation. We only compare to the best models, BERT, BART, and BioBERT, on both MedDialog(EN) and Covid Dataset, because manual evaluation is costly. On fluency, adequacy, and medical knowledge-related criteria *viz.* entity relevance, *MED* outperforms the baseline models, demonstrating consistency with the results of automatic evaluation. All of the kappa values are greater than 0.75, indicating that the annotators agree with each other.

We present a few example conversations predicted by the proposed (*MED*) and baseline models (*BERT*, *BART*, and *BioBERT*) on the test set from MedDialog(EN) corpus and Covid Dataset in Table 10. As seen in the first example, *MED* correctly decodes the response by using context information as well as the correct triplets, such as *(headaches, associated with, migraines), (headaches, co-occurs with, blurring of vision), (headaches, co-occurs with, tingling)* as well as triples formed using the MEP model such as *(pain, medication, ibugesic), (migraine, medication, analgesics), (pain, medication, omeparazole)* as opposed to the BioBERT’s prediction which clearly fails to predict many medical entities. This shows the relevance of enhancing the graph with medications, medical tests and home remedies. The reason for correctly predicting medicines names, such as *omeparazole* and *ibugesic* is the association between *headache* and *migraines* with *pain* as inferred from the triples *viz.(headache, evaluation of, pain), (migraine, associated with, pain)* which is learned by using idea of masking to predict the tail entity i.e pain.

Overall, during evaluation, we found that the presence of medical entities in the predicted response by our proposed *MED* model especially belonging to the medicine, medical test and home remedies increased to 5% and 10% as opposed to the baseline (BioBERT) predictions on MedDialog(EN) and Covid dataset respectively.

We conduct statistical significance tests on our models using statistical hypothesis testing (t-test) at a 5% (0.05) significance level. We used the implementation provided at this link: https://github.com/rtmdrr/testSignificanceNLP to perform the t-test. The test compares the performance of two approaches, A and B, on a given metric M. P-value scores are reported in Table 8 for automatic evaluation and in Table 9 for human evaluation. All the p-values reported in Tables 8 and 9 are less than 0.05 (5% significance level). Hence it shows that our approach is statistically significant. We found that the improvement in the proposed model (MED) over the state-of-the-art models BioBERT, BART, and BERT on the MedDialog(EN) dataset and the Covid dataset, as well as on human evaluation metrics such as Fluency, Adequacy, Emotional Content, and Knowledge Relevance, was statistically significant with 95% confidence (i.e., p-value < 0.05) on both datasets.

**Table 8:** Results of statistical significance test for automatic evaluation metrics.

	MedDialog(EN)						Covid Dataset					
Models	PPL	F1%	BLEU-4	Embedding Average	Vector Extrema	Greedy Matching	PPL	F1%	BLEU-4	Embedding Average	Vector Extrema	Greedy Matching
BERT	1.91E−061	6.03E−062	2.01E−062	2.77E−058	1.39E−017	5.33E−077	2.16E−058	2.82E−026	2.26E−058	2.72E−061	2.51E−016	2.12E−061
BART	2.12E−061	2.26E−058	0.00001	0.0002	0.00001	0.00006	2.82E−062	0.00003	2.11E−058	3.12E−012	1.13E−018	1.22E−012
BioBERT	1.20E−010	3.04E−012	6.04E−022	1.27E−017	1.42E−011	3.33E−012	1.16E−012	1.22E−012	2.12E−08	2.82E−09	4.82E−012	1.12E−012

**Table 9:** Results of statistical significance test for human evaluation metrics.

	MedDialog(EN)			Covid Dataset		
Models	Fluency	Adequacy	Entity Relevance	Fluency	Adequacy	Entity Relevance
BERT	1.21E−011	2.03E−022	1.01E−062	4.27E−018	1.27E−018	1.47E−018
BART	1.12E−011	1.26E−018	1.26E−018	1.56E−018	1.36E−018	2.16E−018
BioBERT	1.20E−010	3.04E−012	6.04E−022	1.27E−017	1.27E−017	1.27E-017

### Ablation study

To analyze the effectiveness of external medical knowledge for medical dialogue generation, we provide an ablation study for our proposed methodology. The results are reported in Tables 6 and 7.

1. *BioBERT*: This setup solely uses the BioBERT-based conversation encoder and decoder to highlight the importance of using augmented knowledge graphs for generating medical dialogue. Table 6 shows that this results in a 5.1 percent decrease in F1 score and a similar drop in human evaluation metrics (Table 7) can also be noted, proving the efficiency of our knowledge assimilation technique in MED.
2. *MED-UMLS:* In this configuration, the triples prepared using the UMLS knowledge base (section “Construction of the medical knowledge graph using UMLS”) are not used; only the triples obtained from the MEP model (section “Construction of the medical entity graph”) are used. Table 6 shows that this results in a 2.2% decrease in F1 score on the MedDialog(EN) dataset, signifying the importance of the triples formed using the entities obtained by the MEP model depicting the importance of local information in order to generate relevant response by the doctor.
3. *MED-MEP:* This ablation model uses only the triples formed using the UMLS knowledge base for medical dialogue generation. Table 6 shows that using only UMLS triples leads to a significant decrease of 13.6% in F1 score on the MedDialog(EN) dataset, which demonstrates that the UMLS knowledge base may overlook some important medical information found in many medical dialogue datasets and instead adds noise into the model and thus supports our motivation to augment the UMLS knowledge graph with more relevant triples.
4. *MED-H*: This experiment is designed to test the effectiveness of our approach for predicting the tail entity. In this module, we try to predict the head entities instead of the tail entities from the augmented knowledge graph for the masked head tokens. The results in Table 6 shows that this leads to a 4.5% decrease in the F1 score and results in a similar performance to the BioBERT model indicating predicting head entities is similar to performing fine-tuning on the dialog dataset and thus demonstrating the effectiveness of our approach of predicting the tail entity given the masked head entities.

**Table 10:** Case study on a sample from the Covid dataset.

Patient	***blurred vision***, ***tingling*** *and numbness in the left hand. intense* ***headache***. ***migraine***? *hi. recently (the past 8 months) i have had this thing happen to me about 4 times. it starts with a* ***blurred vision*** *where i can barely see anything. after that, my left hand gets numb and i get a* ***tingly feeling*** *in it. a few minutes later, the same thing happens to the left lower side of my face. after that i get a terrible* ***headache*** *that will not go away until after 4–5 h. is this just a* ***migraine*** *or should i be worried?*
Doctor	*Hi there, I understand your concern and it is most certain that you are having* ***migraines***. ***Migraines*** *are easily treated in their acute phase and there are medications to prevent these kinds of* ***headaches*** *as well. The triptan group of medications, like* ***sumatriptan***, *can help you block the headache at onset and you should consult a psychiatrist to get the right medication and dosage. I would also think that a beta blocker for the prevention of these kinds of* ***headaches*** *is also going to be helpful. However, consulting a neuro psychiatrist will always be helpful to you. Take care and have a lovely day!*
Triples	(headaches, associated with, migraines), (headaches, co-occurs with, blurring of vision), (headaches, co-occurs with , tingling), (pain, medication, ibugesic), (migraine , medication, analgesics ), (pain, medication, omeparazole), (headache, evaluation of, pain), (migraine, associated with, pain) .
MED	hi, you have mentioned your main problem **headache** for which you have to take **analgesic syrup** or **ibugesic** or **omeprazole**. now as you have described in detail, it is highly likely. there may be some other cause such as **viral fever, drug vomiting**, or some allergies like** drug reaction**, or some medication with body mass. as you are now getting these medications, your condition look like this. still there are no fever episodes so that we just need
BioBERT	hi, thank you for posting your query. i have noted your symptoms. migraine headaches can be associated with migraine headaches or migraine. i appreciate from the history that you have provided. however, the headache is very well from migraine and can not be due to any other disease. in my opinion, you should consult an ophthalmologist for the same. you would need a neurologist to exclude any brain lesion, which can lead to headache
BART	you may have refractive errors which normally occur when the blood vessels in the lower limbs is narrowed. this also occur due to many reasons such as stress, anxiety or anemia, or alcohol intake. you need to take treatment for this which is mainly used to treat the disease. so please do not worry. the refractive error is almost 100 percent in favor of migraine. so, i suggest you see a neurologist for workup to rule out other possible causes. hope this
BERT	hello. thanks for writing to us. the symptoms which are not related to migraine detailed answer : hello, the type of headache, headache and headache can be explained from migraines. if the headache is there which i mean while i recommend avoiding sitting on the inner ear. if your headache persists and is nothing else. in the most common causes are degeneration, numbness in your lips. however you described , it sounds may be because that you might be having

Triples show the corresponding medical knowledge for each patient-doctor utterances.

### Error analysis

Using the generated responses, we conduct a thorough examination of our proposed model and classify the errors it encounters into the following categories:

1. **Non-clinical Response**: We observed around 1% cases where responses as *“hello & welcome to healthcaremagic!”* appear multiple times without any text with medical information. This is due to the fact that using a simple decoder in conjunction with complex context vectors can easily result in non-clinical and generic responses.
2. **Non-Fluency**: We observe around 5% cases of non-fluent outputs. For example, *“hi, i understand your concern to your history and your history. yes, i can help you better, the only way to find your particular diagnosis that you might be suffering from benign tumor of left breast. ... if it is just a simple cyst of the small and in the future i”*. The reason for this is that input and output sequences are truncated because the maximum sequence length for pre-trained models is limited to a fixed length.
3. **Knowledge irrelevancy**: We observe around 5% cases of knowledge irrelevancy. For example, Patient: *“my baby has fever even after giving* ***paracetamol***. *please advise . hello doctor, my four month old baby weighs 7 kg. he has a 100-degree* ***fever*** *since today noon. i had given 4 ml of 125g paracetamol syrup. but the temperature is still at 100.”*; Doctor: *“you can continue giving the 4ml* ***paracetamol*** *syrup, every four to six hours if there is a fever. it will take an hour or two for the temperature to normalize. if fever is nearing 102f, give* ***ibuprofen*** *70mg. it is most likely to be a* ***viral fever***. *so, if the baby is active, you can wait for two days. if you notice any new symptoms, or if the fever persists beyond 48 hours, then consult his previous doctor.”*; Predicted Response: *“don’t worry. with the help of medicine, it is fine. please don’t worry, as such nothing is required. if his symptoms not responding to him, wait and watch.”*. We discovered that, while our proposed model produced an adequate response, it missed many clinical entities that were important. This might be attributed to the fact that since a head entity can have multiple tail entities associated with it and selection of relevant tail entities is still an open problem.

## Conclusion and future works

A virtual doctor uses relevant knowledge graphs to generate clinically correct responses to patient queries. This is critical for the development of a robust and effective medical dialogue system. Previous approaches to integrating knowledge graphs only considered attentive methods to encode them into the current utterance. We proposed a knowledge-driven neural conversational model for medical dialogue generation in this paper. Unlike previous methods, we incorporate the knowledge without using any attention mechanism and by encoding the augmented knowledge graphs alongside the patient’s utterances using the large-scale pretrained language models. We use knowledge from the UMLS database and improve it using medical entity annotation in a semi-supervised fashion to facilitate effective conversation understanding and generation. On the MedDialog testset, our proposed method, MED results in a significant performance improvement of around 5.2 F1 score points and 8.3 BLEU-4 points. On F1 and BLEU-4, MED outperforms the BioBERT baseline by 8.5 and 9.3 points, respectively, on the Covid Dataset. The results on two benchmark medical dataset show masking relevant tokens by jointly utilizing the dialogues and graph based knowledge can successfully enrich entity embeddings based on the dialogue context and hence produce clinically correct and instructive responses. Aside from these, the model’s small misrepresentations are discussed in detail in the error analysis section.

In the future, we aim to make use of commonsense knowledge graphs as well as introduce reward functions to tackle the non-clinical response and non fluency errors for the model. The codes and dataset used to replicate our findings are available at https://github.com/deekshaVarshney/MED.git

## Data Availability

The MedDialog(EN) and CovidDialog dataset analysed in this work are included in these published articles by Zeng et al.^50^ and Yang et al.^59^ and can be downloaded from the links: https://github.com/UCSD-AI4H/Medical-Dialogue-System and https://github.com/UCSD-AI4H/COVID-Dialogue respectively. The extended CovidDialog dataset renamed as the *Covid Dataset* analysed / generated during the current study as explained in section “Covid dataset” are available in the https://github.com/deekshaVarshney/MED.git repository. We have crawled this data from the same websites (https://www.icliniq.com/ and https://www.healthcaremagic.com/) which is an online platform of healthcare services and all the rights reserve to them. The website completely anonymizes the patient’s identity.
